# Effect of Celastrol on Growth Inhibition of Prostate Cancer Cells through the Regulation of hERG Channel *In Vitro*

**DOI:** 10.1155/2015/308475

**Published:** 2015-03-19

**Authors:** Nan Ji, Jinjun Li, Zexiong Wei, Fanhu Kong, Hongyan Jin, Xiaoya Chen, Yan Li, Youping Deng

**Affiliations:** ^1^Medical College, Wuhan University of Science and Technology, Wuhan 430065, China; ^2^The Outpatient Department, The Second Affiliated Hospital, Zhejiang University School of Medicine, Hangzhou 310009, China; ^3^Puren Hospital Affiliated to Wuhan University of Science and Technology, Wuhan 430081, China; ^4^Department of Internal Medicine, Rush University Medical Center, Chicago, IL 60121, USA

## Abstract

*Objective.* To explore the antiprostate cancer effects of Celastrol on prostate cancer cells' proliferation, apoptosis, and cell cycle distribution, as well as the correlation to the regulation of hERG. *Methods.* DU145 cells were treated with various concentrations of Celastrol (0.25–16.0 *μ*mol/L) for 0–72 hours. MTT assay was used to evaluate the inhibition effect of Celastrol on the growth of DU145 cells. Cell apoptosis was detected through both Annexin-V FITC/PI double-labeled cytometry and Hoechst 33258. Cell cycle regulation was examined by a propidium iodide method. Western blot and RT-PCR technologies were applied to assess the expression level of hERG in DU145 cells. *Results.* Celastrol presented striking growth inhibition and apoptosis induction potency on DU145 cells *in vitro* in a time- and dose-dependent manner. The IC_50_ value of Celastrol for 24 hours was 2.349 ± 0.213 *μ*mol/L. Moreover, Celastrol induced DU145 cell apoptosis in a cell cycle-dependent manner, which means Celastrol could arrest DU145 cells in G_0_/G_1_ phase; accordingly, cells in S phase decreased gradually and no obvious changes were found in G_2_/M phase cells. Through transmission electron microscope, apoptotic bodies containing nuclear fragments were found in Celastrol-treated DU145 cells. Overexpression of hERG channel was found in DU145 cells, while Celastrol could downregulate it at both protein and mRNA level in a dose-dependent manner (*P* < 0.01). *Conclusions.* Celastrol exhibits its antiprostate cancer effects partially through the downregulation of the expression level of hERG channel in DU145 cells, suggesting that Celastrol may be a potential agent against prostate cancer with a mechanism of blocking the hERG channel.

## 1. Foreword

Ion channels exist widely in all cells in a variety of physiological functions. Cancer is also associated with ion channel dysfunction. Research [1] recently suggested that some potassium channels (voltage-gated potassium channels, KV) are related to the occurrence and development of malignant tumors, and the relationship between voltage-gated potassium channels and tumor has become a research hotspot. The human EAG gene (human ether-à-go-go related gene, HERG) encodes the HERG protein A subunit of delayed rectifier potassium channel. Researches have found that [2] high expression of HERG protein in tumor cells has a widespread impact on the biological behavior of tumors and is closely related to the differentiation and invasion of tumor cell proliferation and apoptosis [3–5]. There are reports that hERG protein can affect the tumor cell membrane potential in the depolarized state, which is conducive to tumor cell survival, proliferation, and invasion [5]. Therefore, hERG potassium channel will become a promising target for cancer therapy in the selection of specific molecular targeted agents that play an important role in the process. Celastrol (CSL) is one of the main active components extracted from the traditional Chinese medicine* Tripterygium wilfordii*. A general and three-terpene pigment monomer, Celastrol, is found early to have anti-inflammatory and antitumor pharmacological effects on liver cancer, colon cancer, lung cancer, leukemia, cancer of the esophagus, brain cancer, bladder cancer, and so on. Much tumor cell growth and proliferation were inhibited [6, 7]. But its antitumor mechanism has only in recent years seen sporadic reports. Its role in the regulation of hERG potassium channel protein has not been reported. Therefore in this paper, with hERG potassium channel as the molecular target, the effect of different concentration of tripterine on its regulation and tripterine antitumor effect correlation will be investigated.

## 2. Materials and Methods

### 2.1. Drugs and Reagents

Tripterine, molecular formula (C29H38O4), a molecular weight of 450, and purity more than 95%, was purchased from USA Calbiochem company, dissolved in two dimethyl sulfoxide (DMSO), and kept at −20°C, before using DMEM culture medium diluted to final concentration. DMEM medium and fetal calf serum were from the USA Gibco BRL company. Annexin-V/PI apoptosis kits were purchased from Wuhan Boster Engineering Co., Ltd. Rabbit anti-human hERG1 monoclonal antibody was purchased from Sigma company, HRP standard. Sheep anti free, two anti purchased from USA Santa Cruz products. Bio-Rad protein assay kit, TRIzol kit, and ECL lighting kits originated from Sweden Amersham company. RT-PCR kit was purchased from Fermentas company. Primers were synthesized by Shanghai Sangon company.

### 2.2. Cell Lines and Cell Culture

Prostate cancer cell DU145 was obtained from Tongji Medical College, Department of Immunology. Medium contained 10% fetal bovine serum, penicillin 100 IU/mL, and streptomycin 100 g/mL DMEM, at 37°C, 5% CO_2_, and water saturated humidity condition. Every 1~2 days, for a fluid passage, the logarithm growth period of cell activity of more than 98% cells was used in this study.

### 2.3. Effects of* Tripterygium wilfordii* Red MTT Method to Detect the Proliferation of DU145 Cells

Logarithmic growth phase DU145 cell experiments, cells per hole 2 × 10^5^/mL cells were seeded in 96-well plate, adding different concentrations of celastrol (0.25–16.0 *μ*mol/L), with the culture solution containing equal volume DMSO as blank control, each drug concentration group with 3 holes, each hole total volume 200 *μ*L. For 5% CO_2_, 37°C incubator culture 24–72 h, each hole with 5 mg/mL MTT 20 *μ*L reagent, 37°C incubate for 4 h, carefully to absorb the air in culture supernatant, DMSO solution was added to each well of 150 *μ*L, 10 min oscillation, crystallize fully dissolved in the Bio-Rad M450 enzyme labeled each hole optical density were measured in 492 nm wavelength 1.25 meter value (OD), with each experiment repeated 1.273 times, to calculate the inhibitory rate of cell proliferation. Proliferation inhibition rate (%) = (1 − experimental group, control group od/OD) × 100%.

### 2.4. Annexin-V/PI Staining to Detect Apoptosis

Operate according to kit, divided into the experimental group and the control group with single standard. A collection of different concentration of tripterine (1, 2 and 4 *μ*mol/L) DU145 cells and blank treated cells in control group, with 4°C ice cold PBS wash 2 times, and then to 1 × 10^6^/mL cell density weight suspended from 100 *μ*L binding buffer, adding 5 *μ*L Annexin-V and 10 *μ*L PI dye solution, mix gently, light reaction temperature 15 min, add 300 *μ*L of the buffer solution, detection of it within 1 h.

### 2.5. Hoechst 33258 Staining for Detection of Cell Morphology of Apoptosis

The logarithmic growth phase DU145 cell was 5 × 10^5^ cells, join the 6 hole plate with cover glass. Blank group and tripterine IC_50_ 100 *μ*mol/L dosing experiment group, after 24 h incubation, washed two times with PBS, then with fixed liquid (methanol : acetic acid = 3 : 1) fixed 15 min, washed two times with PBS; 37°C Hoechst 33258 staining solution (5 mg/L) staining of 15~30 min, PBS wash two times; neutral resin sheet, fluorescent cell morphology was observed under microscope and photographed. Mirror were found 5 does not repeat, apoptotic cell count per 200 cells, the percentage of apoptotic cells is the apoptosis rate.

### 2.6. The Cell Cycle Was Detected by Flow Cytometry

Collection of the treated DU145 cell was 1 × 10^6^, washing with PBS buffer 2 times, with 70% cold ethanol at 4°C fixed overnight, centrifugation, washing 1 time with PBS, adding 20 *μ*L RNase A at 37°C water bath for 30 min and then adding 300~500 *μ*L PI dye solution mixing and placing 4°C under dark condition for 30 min; the cell cycle was detected by flow cytometry, with red fluorescent wavelength at 488 nm.

### 2.7. Effects of* Tripterygium wilfordii* by Semiquantitative RT-PCR Detection of Red Pigment on the Expression of HERG Gene in DU145 Cells

TRIzol kit was used to extract total cellular RNA synthesis of cDNA, according to the instructions. In the first chain cDNA cells were used as template, PCR reaction. PCR primer was synthesized by Sangon company in Shanghai, of which hERG gene upstream primer was 5′-CAGCGGCTGTACTCGGGCACAG-3′, downstream primer was 5′-CAGAAGTGGTCGGAGAACTC-3′, amplified fragment is 345 bp; 3-glyceraldehyde phosphate dehydrogenase gene (GAPDH) upstream primer is 5′-GATTTGGTCGTATTGGGGCGC-3′, downstream primer is 5′-CAGAGATGACCCTTTTGGCTCC-3′, amplified fragment is 136 bp. The PCR amplification conditions were 95°C denaturing 5 min, 94°C 1 min, 55°C 50 s, 72°C 1 min, cycle 35, 72°C 10 min end reaction. PCR products were detected by 1.5% agarose gel electrophoresis, UV photography, and scanning analysis, the hERG/GAPDH expression of hERG semiquantitative analysis of the level of.

### 2.8. Detection of Western Blot Methods

Different concentrations of tripterine treated DU145 cells and control cells, with cell lysate 100 *μ*L pre-cooling (configuration according to molecular cloning method) the ice cracking 30 min, extraction of total cellular protein, quantitative protein by Lowry method. Conventional adhesive preparation, sampling, protein electrophoresis, and then transferring to the membrane were done. Rabbit anti-human hERG1 monoclonal antibodies were added (1 : 1500), 4°C overnight incubation. Rinse after adding HRP-labeled goat anti-rabbit IgG (1 : 2000), at 37°C with shaking and incubation for 1 h. Finally, ECL chemiluminescence reagent, X-ray exposure and development, analysis of computer software. Each concentration was repeated 3 times, taking the mean measurement results.

### 2.9. Statistical Analysis

Experimental data $\bar{\text{X}}$ ± S, among groups, were compared using* F* test, SPSS 11.5 statistical software analysis.

## 3. Results

### 3.1. Effects of Tripterine on Proliferation of DU145 Cells

**Figure 1:**
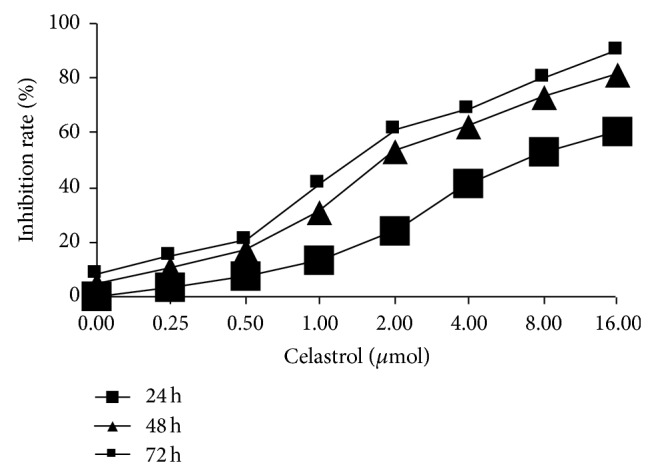
Effects of Celastrol on the proliferation of DU145 cells.

It can be seen from Figure 1, respectively, by 0.25, 0.5, 1, 2, 4, 8, and 16 *μ*mol/L tripterine in DU145 cells after 24~72 h, that cell proliferation activity in different cell groups was lower than that of control cells, but in a concentration less than 1 *μ*mol/L, proliferation effects of tripterine on DU145 cells are small, and when the tripterine concentration reached 1 *μ*mol/L, the proliferation inhibition activity was significantly increased, with significant difference (*P* < 0.05). And, with the increase of tripterine drug concentration and action time, the inhibitory effects of proliferation were enhanced, and an obvious time dose effect relationship is apparent. The 24 h IC_50_ value was 2.349 ± 0.213 *μ*mol/L.

### 3.2. Effects of Tripterine on Apoptosis of DU145 Cells

**Figure 2:**
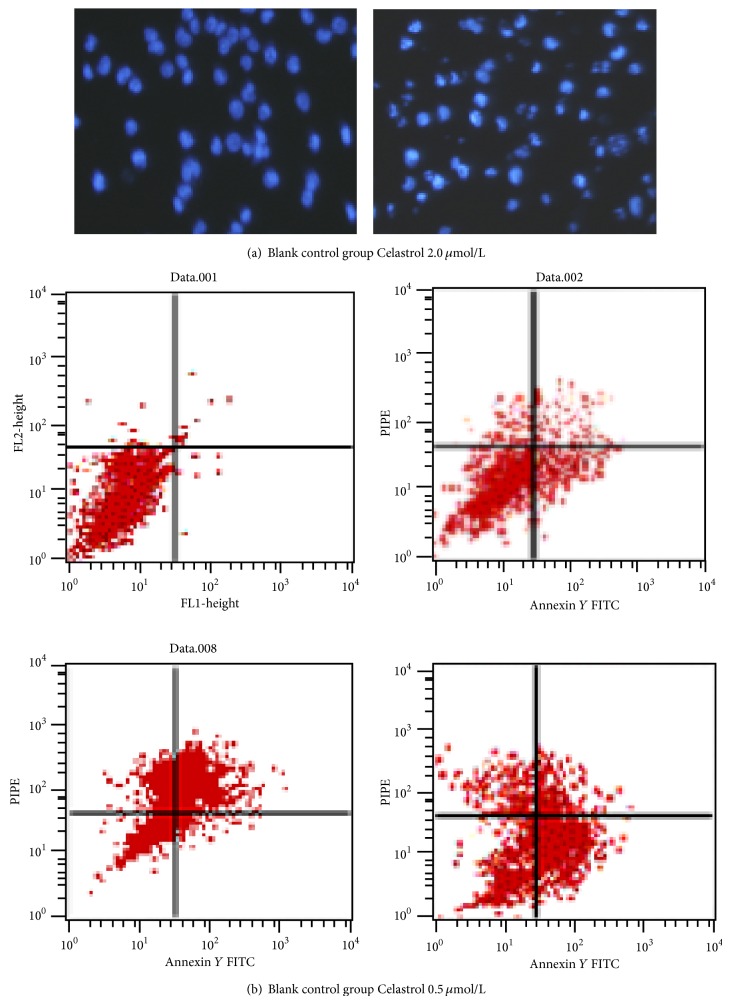
(a) Apoptosis morphological changes of DU145 cells induced by Celastrol with Hoechst 33258 (24 h). (b) Effects of Celastrol on cell apoptosis with Annexin-V FITC/PI assay. Cells were treated with various concentrations of Celastrol for 24 h.

The Hoechst 33258 results can be seen. DU145 cells of 2 *μ*mol/L tripterine acid treatment in typical apoptosis morphological changes are as follows: cytoplasmic density, chromosome condensation, marginalization, nuclear condensation, and formation of apoptotic bodies increased (Figure 2(a)). Then the Annexin-V/PI double staining (Figure 2(b)) quantitative detection results show that, with the increase of tripterine concentration, apoptosis of DU145 cells gradually increases the proportion, respectively, (6.57 ± 0.11)%, (11.02 ± 3.10)%, and (23.23 ± 1.56)% and the control group (2.24 ± 1.08)% in comparison and the difference had statistical significance.

### 3.3. Effects of Celastrol on the Cell Cycle of DU145 Cells

**Figure 3:**
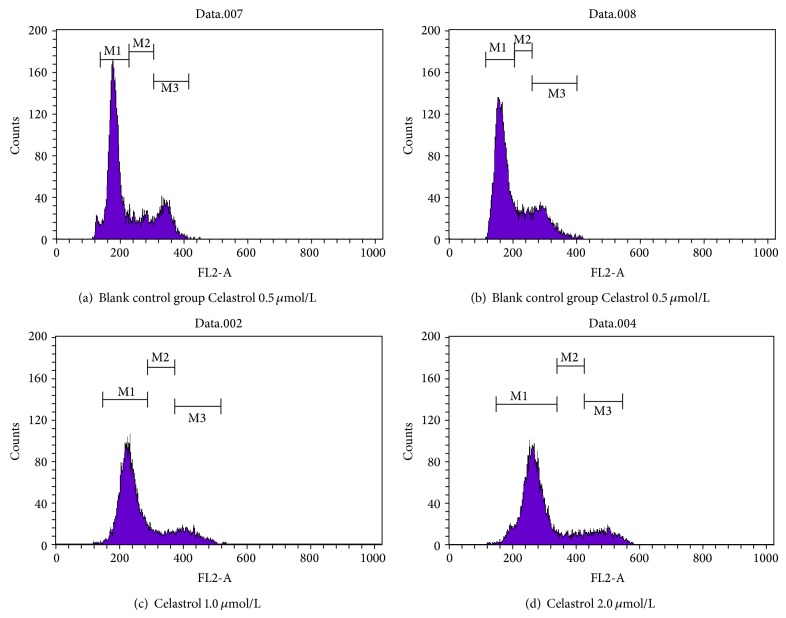
Effects of Celastrol on cell cycle distribution.

With different concentrations of tripterine and the role of DU145 cells after 24 h, the cell cycle distribution has also been changed, as shown in Figure 3. With the increase of tripterine concentration, the percentage of G_0_/G_1_ phase cells increased gradually, followed by (39.95 ± 1.88)%, (40.48 ± 2.34)%, (51.40 ± 1.96)%, and (68.58 ± 2.89)%, while the percentage of cells in S phase was dose-dependently reduced, followed by (49.45 ± 1.67)%, (46.19 ± 1.86)%, (37.67 ± 2.03)%, and (23.29 ± 1.52)%; in contrast, Celastrol effects on G_2_/M cells were not significant (Table 1). This indicates that Celastrol induced DU145 cell cycle arrest occurs in G_0_/G_1_.

### 3.4. Regulatory Effect of Tripterine on DU145 Cells of hERG Potassium Channel Protein

**Figure 4:**
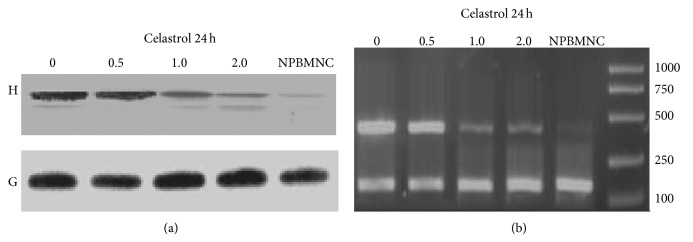
(a) Effects of Celastrol on the expression of hERG protein in DU145 cells and normal mononuclear cells with various concentrations for 24 h. (b) Effects of Celastrol on the expression of hERG mRNA in DU145 cells and normal mononuclear cells with various concentrations for 24 h.

Compared with normal mononuclear cells, the presence of hERG potassium channel protein expression levels was higher in DU145 cells and the 0.5~2.0 *μ*mol/L tripterine was treated for 24 h. The protein expression had a concentration dependent decline, with a statistically significant difference (*P* < 0.05). In order to further clarify the role of* Tripterygium wilfordii* red on the hERG protein, we examined the changes of hERG protein and mRNA content in the level of gene transcription. Similarly, hERG potassium channel protein level of mRNA was dose-dependently downregulated and obviously higher than the mononuclear cells of normal hERG gene expression level (Figure 4).

## 4. Discussion

People have found that many natural preparations, especially in plants and food components, have significant antitumor activity* in vitro* and* in vivo*. In the early 1970s, there were reports of tripterine having anti-inflammatory, analgesic, antioxidant, and antiviral effects, and inducing apoptosis of tumor cells is more positive. Further, to determine the effective components of gambogic acid differently from the general characteristics of anticancer drugs, it can selectively kill tumor cells, but normal hematopoietic cells and heart, liver, kidney, and other organs showed no obvious damage, so it is considered to be a safe and effective antitumor drug [8, 9] for long-term continuous use. The antitumor mechanism has had some scattered reports, but studies in prostate cancer are very rare. In this experiment, cultured prostate cancer cell line DU145 is used as the research object to observe the effect of tripterine on DU145 cell growth inhibition and apoptosis induction and to explore its possible molecular mechanism.

The results show that Celastrol can inhibit the proliferation of DU145 cells and the inhibition is associated with the duration of drug action and drug concentration. At the same time, Celastrol can induce apoptosis of DU145 cells through strong 0.5~2.0 *μ*mol/L tripterine treated for 24 h, the apoptosis rate of DU145 cells increased significantly, and the typical morphological changes of cell apoptosis emerged. The Celastrol-induced apoptosis of cycle arrest effect may be related to induction of closely related events. With the increase of tripterine concentration, the percentage of G_0_/G_1_ phase cells increased gradually and the percentage of cells in S phase decreased gradually and the tripterine had little effect on the percentage of cells in G_2_/M phase. That Celastrol operating mainly by blocking DU145 cells at G_0_/G_1_ phase to have an apoptosis inducing effect has been reported in the literature [10].

Celastrol has become a hot spot of oncology field expression of ion channel proteins in normal and tumor cells. The ion channel proteins in numerous studies, tumor cell lines by hERG potassium channel protein encoded by the hERG gene as selective surface in different tissues and primary tumor cells, and tumor cell proliferation, differentiation, apoptosis, invasion, and sensitivity to chemotherapy are closely related and are considered to be the molecular target in cancer cells more specifically. In general, the hERG gene was only expressed in the early stages of embryonic development and followed by the inward rectifier potassium channel current it was replaced by [11]. HERG potassium channels are voltage gated ion channels typical in mammals. hERG due to the voltage dependence of fast inactivation exhibited strong inward rectification activities and played an important role in maintaining the differentiation and physiological function of normal heart rhythm and neurons [12, 13]. In addition, hERG potassium channel is involved in a variety of ventricular arrhythmias and may be the cause of congenital long QT syndrome (LQTS) which is one of the major virulence factors [14]. As mentioned before, the hERG potassium channel protein is crucial in maintaining the cell resting membrane electric's localization in the polarization state, but new research shows that the proliferation characteristics and cell membrane limited tumor cell depolarization are closely related to the status, suggesting the protein and tumor cell proliferation activity of hERG potassium channel. And successive studies have confirmed that hERG protein was highly expressed in tumor cells and primary cells of various tissues of endometrial cancer, colon cancer, and neuroblastoma derived, while in the normal tissues or cells in the corresponding source, no expression or low expression of [4, 5] was found. In addition, a variety of tumor cells, which include the hERG potassium channel proteins, are also present in prostate cancer cells with high expression [3]. Blockade of the hERG potassium channel proteins by specific agents can inhibit the proliferation and metastasis of tumor cells and the corresponding [15, 16], increasing the sensitivity of tumor cells to chemotherapeutic drugs. At present, hERG potassium channel protein with tumor necrosis factor, integrin receptor, VEGF protein interaction, and active cancer protein [17–20] have been studied. It is not difficult to see that hERG potassium channel is a promising target for cancer therapy. Therefore, in this experiment, hERG gene as a target observe the red Chinese medicine* Tripterygium wilfordii* on DU145 cells of hERG potassium channel protein regulation. The results show that, compared with the mononuclear cells of normal control, the expression of hERG potassium channel protein levels rises to higher DU145 cell memory, while the normal mononuclear cells hardly show expression. For the effect of tripterine intervention, the protein and gene expression levels were concentration-dependent underground tune. The effect of tripterine on proliferation of DU145 cells and the intracellular expression of hERG potassium channel are closely related. Although confirmed in tumor cells, inhibition of hERG potassium channel protein expression can inhibit the proliferation of tumor cells, inducing tumor cell apoptosis. Tripterine is expected to become the hERG potassium channel protein inhibitor of a new generation.

Effects of hERG potassium channel protein on the biological behavior of the tumor that can inhibit the growth of tumor cells by inhibiting the expression or channel current of IhERG and promote tumor cell differentiation or apoptosis, reduce its invasiveness. There lays a good foundation for tumor targeting therapy and drug screening.

## Figures and Tables

**Table 1:** Effects of Celastrol on cell cycle distribution and early apoptosis (*n* = 3, ±S).

Celastrol	Cell cycle (%)			Apoptosis rate (%)
(*μ*mol/L)	G_1_/G_0_	S	G_2_/M	Sub-G_1_
0 (control)	39.95 ± 1.88	49.45 ± 1.67	10.60 ± 1.33	2.88 ± 0.33
0.5	40.48 ± 2.34	46.19 ± 1.86	13.33 ± 1.84	1.69 ± 0.24
1.0	51.40 ± 1.96^**^	37.67 ± 2.03^**^	10.93 ± 0.98	3.25 ± 0.78
2.0	68.58 ± 2.89^**^	23.29 ± 1.52^**^	8.13 ± 1.02	13.77 ± 2.15^**^

^**^ *P* < 0.01 versus control group.
